# Pulsed laser diode excitation for transcranial photoacoustics

**DOI:** 10.1364/BOE.570037

**Published:** 2025-08-15

**Authors:** Maxim N. Cherkashin, Jan Laufer, Thomas Kirchner

**Affiliations:** 1 Photonics and Terahertz Technology, Faculty of Electrical Engineering and Information Technology, Ruhr University Bochum, Universitätsstrasse 150, 44780 Bochum, Germany; 2 Medical Physics, Institut für Physik, Martin-Luther-Universität Halle-Wittenberg, Von-Danckelmann-Platz 3, 06120 Halle (Saale), Germany

## Abstract

Photoacoustic (PA) imaging of deep tissue tends to employ Q-switched lasers with high pulse energy to generate high optical fluence and therefore high PA signal. Compared to Q-switched lasers, pulsed laser diodes (PLDs) typically generate low pulse energies. In PA applications with strong acoustic attenuation, such as through human skull bone, the broadband PA waves generated by nanosecond laser pulses are significantly reduced in bandwidth during their propagation to a detector. As high-frequency PA signal components are not transmitted through the skull, we propose not to generate them by increasing the excitation pulse duration. Because PLDs are mainly limited in their peak power output, an increase in pulse duration linearly increases pulse energy and, therefore, PA signal amplitude. Here, we show that the optimal pulse duration for deep PA sensing through thick human skull bone is far higher than in typical PA applications. Counterintuitively, this makes PLD excitation well-suited for transcranial photoacoustics. We show this in PA sensing experiments on *ex vivo* human skull bone.

## 1. Introduction

Photoacoustic (PA) waves are generated by pulsed light excitation of optical absorbers. This enables centimeter-deep acoustic imaging of optical absorption [1], arising from chromophores, such as oxy- and deoxyhemoglobin. PA imaging of the brain is the subject of intense research [2], as clinical applications of PA brain imaging promise functional assessment of perfusion and blood oxygenation that could assist in time-critical diagnosis of brain injury such as stroke. To date, PA brain imaging has been demonstrated non-invasively in small animals [3] and experiments using *ex vivo* skull [4–7]. *In vivo* PA brain imaging in large animals [8] or humans [9] has only been demonstrated following a craniectomy.

For deep PA imaging, high excitation pulse energies are generally desirable to achieve a maximized signal-to-noise ratio (SNR). These pulse energies are typically achieved using Q-switched excitation lasers. At fixed wavelengths, like 1064 nm, Q-switched lasers can reach pulse energies up to ∼
 1 J, at a pulse repetition rate (PRR) of 1 to 100 Hz. For a tunable near-infrared wavelength, optical parametric oscillators (OPO) are used, which provide pulse energies of tens of mJ. For *in vivo* PA imaging, applicable PRRs and pulse energy are limited by the maximum permissible exposure (MPE) of skin. The pulse durations of Q-switched lasers are typically a few nanoseconds, in some cases tens of nanoseconds [9], which results in a broadband PA response, up to tens of MHz.

The acoustic attenuation of PA waves in skull bone is highly frequency dependent [5,10,11]. The transmitted acoustic power spectrum is cut off at 1 to 3 MHz, with higher thickness and porosity leading to lower cut-off frequencies and more attenuation. Frontal bone is representative of high thickness, high attenuation cranial bone. For PA imaging through such bone, the generated acoustic spectrum can be limited to a few MHz, which should allow for at least an order of magnitude longer PA excitation pulses.

Increasing pulse duration will not directly translate to an increase in pulse energy, and thereby SNR, when using typical Q-switched lasers. However, pulsed laser diode (PLD) arrays are mainly limited by the peak power output of the laser diodes and less so by pulse energy. In quasi-continuous wave (QCW) operation, the duty cycle of PLDs is limited to around 0.1 % to 1 %, allowing the selection of a wide range of pulse durations. Increasing PLD pulse duration can proportionally increase pulse energy at the cost of acoustic power at higher acoustic frequencies [12] – but ideally only in a frequency range which will not penetrate skull bone anyway.

PLDs were first proposed as viable PA imaging excitation sources by Allen *et al.* in 2005 [12,13] and employed for *in vivo* experiments shortly thereafter [14]. During the last two decades, these sources have not seen widespread adoption in PA imaging [15]. However, QCW PLD stacks are well suited as a pump source for YAG lasers, which led to development of even higher power diodes, especially at 808 nm and 940 nm [16]. Using these PLDs for PA imaging enabled highly integrated, potentially multi-wavelength illumination [17]. These higher power laser diodes deliver pulse energies in the mJ range, leading to PA imaging research prototypes [17,18]. Compared to Q-switched excitation sources (see Table 1), currently available PLD stacks feature high PRRs [19], a low cost and small footprint, and are generally used with longer pulse durations. Even though their pulse energies are significantly increased compared to earlier PLDs, they are still orders of magnitude lower than those of the highest-energy Q-switched laser sources.

**Table 1.** Comparison of typical current PA imaging excitation laser systems.

	Q-switched	with OPO	PLD stacks
Pulse energy E	200 – 1000 mJ	10–50 mJ	0.1 – 8 mJ
Maximum PRR	1 – 100 Hz	10 – 100 Hz	200 – 6000 Hz
Pulse duration τ	2 – 20 ns	2 – 20 ns	30 ns – QCW
Wavelength	fixed	tunable	fixed, multiple wavelengths
Cost	<100 k€	>100 k€	≈ 10k€
Footprint	moderate-high	high	low

The combination of low pulse energy and long pulse durations produced by PLDs make them generally less useful for PA imaging than Q-switched lasers. However, specific PA imaging applications could benefit, especially applications with high PRR requirements, limitations in maximum permissible exposure (MPE) or high acoustic attenuation, leading to reduced acoustic bandwidth.

Similarly, PA excitation using light emitting diode (LED) arrays [20] has started to be used. Here, maximum pulse energies are in the order of 0.1 mJ (at 1 kHz PRR) – an order of magnitude less than PLD stacks.

Acoustic attenuation is a limiting factor in achievable depth per resolution across PA imaging modalities [21]. In practice the detectable PA signal bandwidth is typically around 50 MHz for PA microscopy, and 5 MHz for PA tomography in centimeter depths [21,22]. The acoustic attenuation of human skull bone limits the bandwidth of transmitted photoacoustic signals to typically less than 2 MHz [5]. Longer excitation pulse durations lead to PA waveforms of lower bandwidth which, in bandwidth-limiting imaging applications, will still lead to the same transmittable bandwidth – without causing any loss in resolution.

Increasing PLD pulse durations to match the generated acoustic frequency spectrum to the acoustic attenuation in soft tissue has been described theoretically [12,13]. Due to even higher acoustic attenuation in skull bone than in soft tissues, transcranial PA allows for even longer pulses. PLDs can provide these longer pulse durations while also increasing PLD pulse energy and thereby generated PA signal amplitude.

PLD emitters operate at a fixed wavelength, though multiple such emitters can be readily combined to allow for multispectral PA imaging, e.g. functional blood oxygenation measurements [18,23]. This enables multispectral systems to be built at comparatively low cost and footprint. In contrast, available wavelengths for high power Q-switched lasers are limited, with 1064 nm Nd:YAG lasers being the predominant PA imaging excitation laser. Lasers emitting at different wavelengths, e.g. alexandrite and ruby lasers, are rarely used and generally have lower power. Addition of harmonic generators and optical parametric oscillators (OPO) to Nd:YAG lasers offers a wide range of excitation wavelengths but at the cost of pulse energy; and also adds significant cost, complexity and footprint.

In this work, we examine the effect of the duration of excitation pulses generated by different laser sources on PA signals transmitted through thick *ex vivo* human skull bone. We demonstrate that the optimal excitation pulse duration for transcranial PA sensing is two orders of magnitude higher than typical PA excitation pulse durations. We discuss the suitability of PLDs as excitation sources for transcranial PA imaging and sensing.

## 2. Materials and methods

To study the effect of excitation pulse duration and pulse shape on transcranial PA signals, the following three excitation lasers were used:

(1) a Q-switched laser typical for PA imaging,
(2) a constant energy PLD system (CE-PLD), relatively short pulsed,
(3) a constant power PLD system (CP-PLD), custom-made for wider variation in pulse duration.

As excitation source (1) we used a Nd:YAG laser (Quanta-Ray PRO-270-50, Spectra-Physics Lasers, Santa Clara, USA) at 1064 nm. Pulse durations were varied between 25 and 245 ns by adjusting the pump flash lamp voltage. The pulses were attenuated to obtain pulse energies of around 1 mJ.

The two PLD systems are based on vertical diode laser stacks emitting at a wavelength of 808 nm. Both have a small size ( ∼
 1 L), compared to the Q-switched laser (>100 L). The CE-PLD system is a commercial system (QD-Qxy10-IL, Lumibird, France) designed for PA imaging [25,26] with a constant 1 mJ pulse energy, up to 2 kHz pulse repetition rate (PRR) and variable pulse duration between 35 ns and 63 ns. The CP-PLD system (PULS Direct Diode, Monocrom S.L., Barcelona, Spain) was custom-made and is operating at a maximum power of 3.1 kW. It is designed to emit a wide range of pulse durations (100 ns to 700 ns) yielding pulse energies between 0.3 to 2.1 mJ at PRRs of up to 200 Hz. The pulse duration and PRR of the system are limited by the laser diode driver electronics and heat management. The output of the CP-PLD has a divergence angle of 10 degrees on the slow axis and 30 degrees on the fast axis. The output beam of the CE-PLD has a slow axis divergence angle of 10 degrees and a fast axis divergence collimated by a microlens array, from 35 degrees down to 3 degrees.

**Fig. 1.**
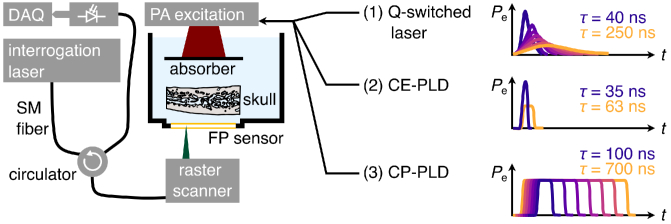
Schematic of the experimental setup. A planar absorber is illuminated by one of three photoacoustic (PA) excitation sources: (1) a Q-switched laser, (2) a pulsed laser diode (PLD) system with constant energy (CE), (3) a PLD system with constant power (CP). The generated PA wave is measured using a planar FP sensor, interrogated by a PA tomography raster scanning setup similar to Zhang *et al.* [24] – using a narrow tunable laser as input and measuring the output voltage of a fast photodiode with a data acquisition (DAQ) card. An *ex vivo* human frontal bone sample is placed in between the FP sensor and the PA source for acoustic transmission measurements. The shape and duration τ
 of the excitation pulse is varied as illustrated by the time-excitation-power curves $P_{\text{e}}(t)$ on the right.

The experiments were conducted in transmission geometry in a water bath with sensor, skull and PA source placed along a common axis (see Fig. 1), similar to previous work [5]. Transmission geometry, in contrast to the reflection geometry applied in most imaging applications (excitation and PA detection from the same side), was chosen to ensure a well-defined PA source that does not change its shape due to the presence of skull bone. The only variation in PA waveform will be due to the variation in pulse duration, with a constant size and shape of the PA source. This was chosen in order to investigate the acoustic effects. The investigation of optical attenuation in skull bone was not subject of this study, because variations in pulse duration will have no effect on light propagation for nanoseconds to CW pulse durations with skin safe fluence. Near infrared light attenuation in skull bone is similar to soft tissue [27–29], with high scattering and low absorption within the bone itself. Light attenuation in skull *in vivo* would be dominated by blood absorption.

To ensure broadband acoustic detection, a planar Fabry-Perot (FP) sensor with bandwidth of 36 MHz and a flat frequency response [30] is used. The PA response of the sensor is measured with a PA tomography raster scanner setup similar to Zhang *et al.* [5,24] using a CL band (1570 nm) interrogation laser (Tunics T100S, Yenista Optics, Lannion, France). PA waves were generated by illuminating a planar absorber (consisting of a thin film of black acrylic paint on an acrylic glass block) with the output of each excitation laser. The absorber was illuminated by a fixed 5 mm diameter Gaussian spot using the Q-switched laser; a fixed 5 mm ×
 5 mm square spot using the CE-PLD; and with a fixed 1 cm ×
 3 cm field for the CP-PLD system with a divergent beam. The absorber was placed in a water bath, parallel to the FP sensor, at a distance of 3 cm from the sensor. The pulse energy was measured using a beam sampler and a pyroelectric sensor (Ophir PE25-C, Ophir Optronics, North Andover, USA). Pulse duration and shape were measured with a fast silicon photodiode (UPD-500-SP, Alphalas, Göttingen, Germany).

In order to measure transcranial PA signals resulting from variable duration PLD PA excitation pulses, an *ex vivo* frontal bone sample was inserted into the transmission path between PA source and FP sensor (Fig. 1). The skull sample [31] consisted of frontal skull bone with an average thickness of 8 mm, obtained from a 70 year old male body donor. Written informed consent for general scientific investigation was given by the body donor. We chose this as a representative example of thick skull bone (frontal cranial bone has a typical thickness of 6 to 9 mm [32]) with strong acoustic attenuation [5]. Such *thick* skull bone – i.e. cranial bone consisting of Diploë sandwiched between cortical bone layers – makes up the majority of the human skull. We measured through the center position of the right frontal cranial bone sample to ensure that no acoustics bypasses the bone sample. To obtain reference measurements, additional PA signals transmitted through degassed water only (without skull) are recorded with each excitation laser.

## 3. Results

We investigated the effect of pulse duration on the acoustic power density spectra of PA signals. Q-switched lasers are typically used for PA excitation with a fixed and short pulse durations. To generate close to Gaussian pulse shapes with variable pulse durations, we increased the pulse duration of a typical Q-switched Nd:YAG laser as described in section 2. All measurements are provided as Open Data (see Data Availability Statement).

**Fig. 2.**
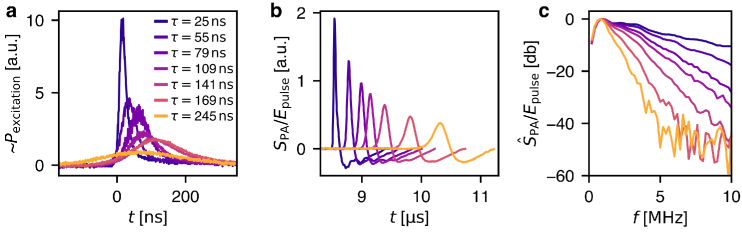
PA excitation with a Q-switched laser with full width at half maximum pulse durations τ
 of 25 to 245 ns. Reference measurement after propagation through 1 cm of water only (without skull bone). **a** Pulse shapes as measured with a fast photodiode. **b** PA waveforms $S_{PA}$ normalized for pulse energy E . **c** Acoustics power spectra – Fourier transforms of the PA signals ${\hat{S}}_{PA}$ , normalized for pulse energy E .

Figure 2(a) shows the time-resolved excitation power for pulses of the Q-switched laser, measured from a reflection off a beam sampler. Figure 2(b) shows the PA signals detected by the ultrasound sensor after propagation through 1 cm of water. The acoustic power spectra of the detected PA signals are shown in Fig. 2(c). They are Fourier transforms of the bipolar energy-normalized waveforms as shown in panel b. As expected, increasing the pulse duration τ
 decreases the bandwidth of the generated photoacoustic waveform. Figure 2(c) shows this behavior for Gaussian pulse shapes, which is in agreement with previous *in silico* results [12].

**Fig. 3.**
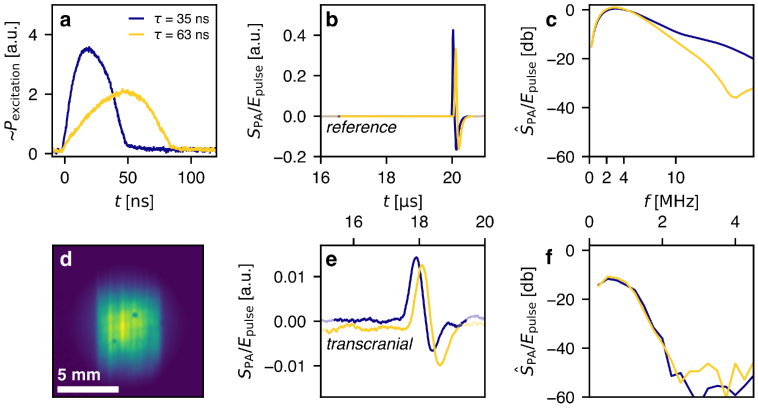
Photoacoustic (PA) measurements of a thin, planar absorber using the constant energy pulsed laser diode (CE-PLD) system. **a** Pulse shape of the PA excitation pulses with pulse durations τ
 . **b** PA reference waveforms $S_{PA}$ for pulse durations of τ
 of 35 ns and 63 ns. **c** Acoustic power density spectra of the reference waveforms $S_{PA}$ (Fourier transforms ${\hat{S}}_{PA}$ ). **d** Maximum intensity projection (MIP) along the acoustic axis of a 3D PA image of the planar absorber, reconstructed from the reference measurements. Single PLD bars collimated by microlenses can be distinguished. **e** PA waveforms measured through thick frontal cranial bone and **f** the corresponding acoustic power density spectra of the transcranial waveforms.

Using the CE-PLD source we included transcranial PA sensing in the experiment. PA waveforms were again excited in a planar thin film absorber with pulses shown in Fig. 3(a). Fig. 3(d) shows the reconstructed initial pressure field in the absorber, illustrating the spatial distribution of the pulse energy on the planar absorber. The heterogeneity of the beam profile results from the collimation by a microlens array. Fig. 3(b)&(e) shows PA waveforms generated using the longest (63 ns) and shortest (35 ns) pulse durations provided by the CE-PLD system. The signals are normalized with the measured pulse energy. The measured pulse energies were in fact similar (0.93 mJ and 0.96 mJ). The PA waveforms as shown in figure Fig. 3(b)&(e) were measured at the center of the FP sensor, on the acoustic axis. In this figure, 400 PA waveforms were averaged to increase SNR as the FP sensor had a relatively low sensitivity.

The PA measurement are performed in transmission with and without the skull sample. In the reference measurements (without skull), power spectra for varying excitation pulse durations differ only at frequencies above 5 MHz, affecting the reference PA signal amplitude as seen in Fig. 3(b). The variation between the power spectra of the transcranial PA signals is insignificant (Fig. 3(f)), as virtually all acoustic power above 2 MHz is attenuated in frontal bone. It is apparent that variations in pulse duration for relatively short durations (up to 63 ns) have negligible effect on transcranial PA signals.

**Fig. 4.**
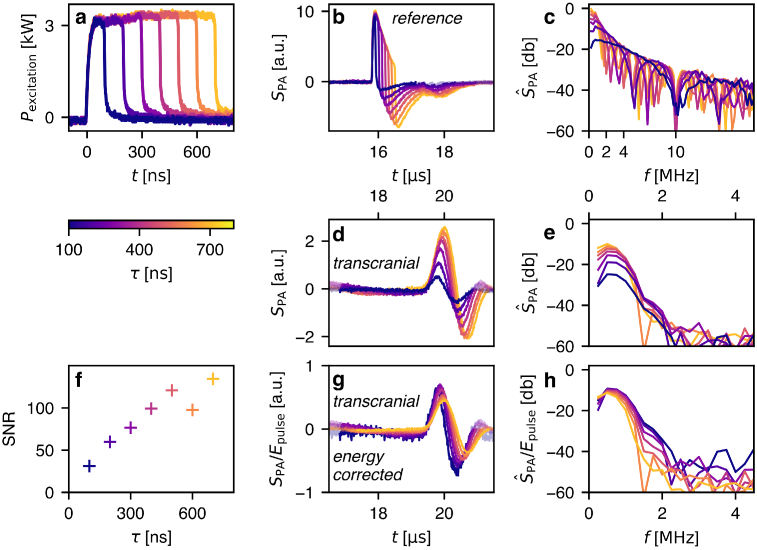
PA transmission measurements through cranial bone using constant power pulsed laser diode (CP-PLD) excitation of varying excitation pulse duration τ
 , ranging from 100ns to 700ns in increments of 100ns . **a** pulse shape measured using a fast photodiode. **b** Reference PA signals, and **c** corresponding acoustic power spectra ${\hat{S}}_{PA}$ . **d** Raw transcranial PA signals, **e** their acoustic power spectra ${\hat{S}}_{PA}$ , and **g&h** the signals and spectra after pulse energy normalization. **f** Transcranial signal-to-noise ratio (SNR) as a function of pulse duration τ
 .

To investigate the effects of longer pulse durations, we used the CP-PLD system with up to 700 ns pulses. As shown in Fig. 4(a), the CP-PLD system was driven at its maximum rated output power, producing square excitation pulses with nearly constant power. The rise and fall times of the square pulses were constant.

Figure 4(b) shows PA waveforms detected after transmission through water. Their respective power spectra are shown in Fig. 4(c). The transcranial PA waveforms, recorded after introducing the *ex vivo* skull sample in the transmission path, are shown in Fig. 4(d), with their respective power spectra in Fig. 4(e). The pulse-energy-normalized transcranial PA waveforms and their power spectra are shown in Fig. 4(g) and Fig. 4(h) respectively. The reference PA waveforms shown in Fig. 4 are averaged over 400 pulses at a pulse repetition rate of 200 Hz. The transcranial PA waveforms are averaged over 2000 pulses in order to visualize all waveforms clearly (including the τ
=100 ns waveform with the lowest pulse energy of 0.3 mJ). In this experiment, the divergent beam of the CP-PLD resulted in a lower fluence at the target absorber and therefore yielded lower amplitude PA waveforms compared to the CE-PLD.

With increasing pulse duration up to 500 ns, the transmitted acoustic power (Fig. 4(e)) increases over the entire frequency range. For pulse durations longer than 500 ns less acoustic power is generated at frequencies of 2 MHz and below.

## 4. Discussion

The reference measurements, performed without skull bone, are consistent with previously described and theoretically expected behavior of long duration PA excitation pulses [12,13]. Increasing the pulse duration of a square pulse (with constant rise/fall times and constant power, see Fig. 4(a)) does not generally lead to an increase of PA signal peak amplitudes but extends the duration of the PA waveform (see Fig. 4(b)) when observed with a sufficiently broadband detector.

In transcranial PA measurements, skull bone acts as a higher-order low-pass filter for the PA waveform, while acoustic attenuation in soft tissue is far less frequency dependent. The transcranial PA signal amplitude increases with pulse duration if the power of the source is constant (Fig. 4(d)). The acoustic power transmitted through the frontal bone (Fig. 4(e)) increases over the full spectrum up to a pulse duration of τ
=400ns . For τ
⪆
600ns the power spectrum exhibits stronger attenuation between 1 and 2 MHz. A pulse duration increase up to ≈
400ns shows no reduction on PA signals transmitted through sufficiently thick skull bone, even when correcting for pulse energy (cf. Figure 4(g)). However, matching the pulse duration to the acoustic attenuation of the tissue does not by itself lead to an increase in SNR if the pulse energy is unchanged – this can be seen in both Figs. 3 and 4(h). Because PLD sources are limited in their maximum power, longer pulse durations increase their maximum pulse energy. The increase in SNR shown in Fig. 4(f)) is therefore solely due to the linear increase in pulse energy as a function of pulse duration. This increase in SNR is linear up to a band limiting pulse duration. For the *ex vivo* skull sample used in this study this pulse duration is approximately 400 to 500 ns, without losses in any band of the acoustic spectrum. As the acoustic spectrum is not influenced by these increased pulse durations, PA imaging applications would not suffer from decreased resolution. *In vivo* skull is expected to cause similar acoustic attenuation. While skull thickness and therefore attenuation varies, the frontal bone sample used had a thickness which was representative of typical human skull bone surrounding the brain. In adult human skull generally, only the temporal bone is significantly more transparent to ultrasound – such thin bone constitutes only a small part of the skull.

Considering excitation pulses on the order of hundreds of nanoseconds, current PLD stack systems can yield a PA imaging SNR similar to OPO excitation lasers, given the same acquisition duration. To give an example using typical lasers, we can compare a PLD with an OPO laser at the same wavelength (i.e. 808 nm). PLD systems with pulse durations longer than 100 ns can deliver 5 mJ pulses with a PRR of 3 kHz. OPO lasers can deliver pulse energies of approximately 30 mJ at a pulse repetition rate (PRR) of 100 Hz. Averaging 30 acquisitions will yield an acquisition rate of 100 Hz, such a PLD source achieves an SNR equivalent to a typical OPO system at an order of magnitude lower cost, and at orders of magnitude smaller footprint in terms of scale and power consumption – making PLDs much more mobile and easy to integrate in medical devices. This reduced footprint at an equivalent SNR to OPO systems makes PLD excitation interesting for clinical translation.

Furthermore, pulse energies of PLD stacks are stable in pulse energy over long acquisitions, with a typical standard deviation of less than 1%. This is useful for functional and quantitative PA imaging, where pulse energy variations are a source of estimation error.

A general limiting factor for imaging depth and SNR is the excitation laser fluence on the tissue surface, which is often limited by the maximum permissible exposure (MPE) [33], defined in terms of pulse energy per skin surface area and dependent on pulse duration. The longer pulses of PLD excitation for transcranial photoacoustics allow for a safer energy delivery and lead to a higher MPE on skin. I.e. for pulse durations τ
=1ns to 100ns , MPE $:=C_{A}\cdot 20\;{\text{mJ/cm}}^{2}$ (with CA=102(λ
[μ
m]−
0.7) in the wavelength λ
 range of 700 nm to 1050 nm) leading to an MPE of 33 mJ/cm^2^ (at λ
=808nm and τ
≤
100ns ). For longer pulses with 100ns<τ
<10s , MPE :=CA⋅
τ
1/4⋅
1.1J/cm2 . Increasing the pulse duration to τ
=500ns leads to an increase in the MPE to 49 mJ/cm^2^. However, for laser exposure of more than 10 s (up to $3\cdot 10^{5}\;\text{s}$ ), the delivered average power is the relevant limit, with $\text{MPE}:=C_{A}\cdot 0.2{\text{W/cm}}^{2}$ , $\text{MPE}(808\;\text{nm})=340\;{\text{mW/cm}}^{2}$ . This effectively limits the maximum PRR.

In addition, PA detection with high sensitivity for very low-frequency ultrasound is needed to make use of long-pulsed PLD excitation, as these low-frequency acoustic components in the PA signal increase linearly with PLD pulse duration. Low-frequency sensitive PA sensors would result in a bandwidth-matched detection to take full advantage of PLD excitation.

We can conclude that increasing PLD excitation pulse duration to hundreds of nanoseconds, increases the SNR of PA signals transmitted through thick cranial bone, because the increase in pulse duration is accompanied by an increased pulse energy.

## Data Availability

Data underlying the results presented in this paper are Open Data at [34].
